# Degradation of Aflatoxin B_1_ by a Sustainable Enzymatic Extract from Spent Mushroom Substrate of *Pleurotus eryngii*

**DOI:** 10.3390/toxins12010049

**Published:** 2020-01-14

**Authors:** Maria Teresa Branà, Lucrezia Sergio, Miriam Haidukowski, Antonio F. Logrieco, Claudio Altomare

**Affiliations:** Institute of Sciences of Food Production, National Research Council (CNR), Via G. Amendola 122/O, 70126 Bari, Italy; mariateresa.brana@ispa.cnr.it (M.T.B.); miriam.haidukowski@ispa.cnr.it (M.H.); antonio.logrieco@ispa.cnr.it (A.F.L.); claudio.altomare@ispa.cnr.it (C.A.)

**Keywords:** *Pleurotus eryngi*, king oyster mushroom, spent mushroom substrate, crude extract, aflatoxins, laccase, feed detoxification

## Abstract

Ligninolytic enzymes from white-rot fungi, such as laccase (Lac) and Mn-peroxidase (MnP), are able to degrade aflatoxin B_1_ (AFB1), the most harmful among the known mycotoxins. The high cost of purification of these enzymes has limited their implementation into practical technologies. Every year, tons of spent mushroom substrate (SMS) are produced as a by-product of edible mushroom cultivation, such as *Pleurotus* spp., and disposed at a cost for farmers. SMS may still bea source of ligninolytic enzymes useful for AFB1 degradation. The in vitro AFB1-degradative activity of an SMS crude extract (SMSE) was investigated. Results show that: (1) in SMSE, high Lac activity (4 U g^−1^ dry matter) and low MnP activity (0.4 U g^−1^ dry matter) were present; (2) after 1 d of incubation at 25 °C, the SMSE was able to degrade more than 50% of AFB1, whereas after 3 and 7 d of incubation, the percentage of degradation reached the values of 75% and 90%, respectively; (3) with increasing pH values, the degradation percentage increased, reaching 90% after 3 d at pH 8. Based on these results, SMS proved to be a suitable source of AFB1 degrading enzymes and the use of SMSE to detoxify AFB1 contaminated commodities appears conceivable.

## 1. Introduction

Aflatoxins (AFs) are mycotoxins produced mainly by fungi of the genus *Aspergillus*. Aflatoxin B_1_ (AFB1) is the most harmful among the Afs and exhibits extremely high hepatotoxic, mutagenic, and carcinogenic effects on humans and animals [1]. AFB1 has been classified by the International Agency for Research on Cancer [2] as a Group 1 substance, that is of proven carcinogenicity to humans. Many agricultural commodities such as groundnuts (peanuts), corn, sorghum, rice, spices, nuts, and several other cereals are subjected to infestation by aflatoxigenic molds and thereby contamination with AFB1 [3,4]. Human exposure to AFB1 can result directly from the ingestion of contaminated food or indirectly from the consumption of products from animals fed with contaminated feed [5]. According to the Rapid Alert System for Food and Feed (EU RASFF) reports [6], in the last ten years, mycotoxins, particularly AFB1, have been the first cause of rejection of imported products, leading to severe economic losses, especially for developing countries [7]. Currently, there are no completely effective methods for the prevention of pre-harvest contamination, and decontamination is often ineffective or not economically sustainable. Extensive research has been carried out for the control of mycotoxins into feed and foodstuffs by using heat, solvents, and chemicals, but these methods may compromise the nutritional, sensory, and functional proprieties of the products [8]. 

Recently, the biological degradation of AFB1 has been considered as a feasible technology for remediation of AFB1-contaminated products [9]. Many studies of AFB1 degradation by living microorganisms have been conducted, especially with bacteria [10,11,12,13,14,15,16,17] and with white- and brown-rot fungi [18,19], as well as with ligninolytic enzymes purified from these fungi [20]. Ligninolytic enzymes catalyze the formation of radicals by oxidation, and thus destabilize the molecular bonds. They have an extremely low specificity of substrate, being able to degrade lignin as well as a wide range of other polycyclic aromatic hydrocarbons [21]. Enzymatic degradation offers several advantages due to the specific and permanent action, which results in alteration of the toxin structure and its transformation into non-toxic or less toxic molecules, such as its hydroxy derivative aflatoxicol [22]. Yehia [23] has shown that an Mn-peroxidase purified from the culture filtrate of *P. ostreatus* was able to detoxify up to 90% of AFB1. Alberts and colleagues [24] reported the first evidence of a significant correlation between laccase activity and AFB1-degradative capability of *P. ostreatus* isolates. Most recently, a purified laccase isoform from *P. eryngii* in combination with laccase mediator systems was reported to be able to degrade up to 73% of AFB1 [25]. However, the high cost of enzymespurification has limited their implementation into applied technologies for practical control of AFB1 in the food and feed chains. 

Recently, due to the growing demand for protein-rich food, a significant increase in the industrial production of edible mushrooms has occurred. Worldwide, the top three mushroom cultivated species are, in order of production: white button mushroom (*Agaricus bisporus*), shiitake (*Lentinula edodes*), and oyster mushroom (*Pleurotus* spp.). The genus *Pleurotus*, with five or six cultivated species, accounts for 19% of world production [26] and cultivation that spans from temperate to tropical areas. The species *P. eryngii* is of special interest, due to a remarkable flavor, high nutritional value, and medicinal proprieties [27,28], and is the most extensively cultivated mushroom in the southern Italy region of Apulia. *Pleurotus* spp. possess a well-developed ligninolytic enzyme system, particularly effective in the degradation of both lignin and different aromatic compounds [29]. These mushrooms are also easily grown on substrates that do not require any special pretreatment and that consist primarily of agro-industrial and forestry waste materials [30]. 

It has been calculated that every kilogram of mushrooms produced generates about 1–2 kg of dry spent mushroom substrate (SMS) [31], which is disposed as waste at a cost for the farmer. After harvest, the remaining SMS is rich of extra-cellular enzymes, such as lignin peroxidase (LiP, E.C. 1.11.1.14), Mn-dependent peroxidase (MnP, E.C. 1.11.1.13), and a copper-containing oxidoreductase, laccase (Lac, E.C. 1.10.3.2) [32]. Therefore, some research efforts have been oriented toward the use of SMS as a promptly available and inexpensive source of ligninolytic enzymes [33,34,35,36] and a few technological applications have been proposed [37,38,39]. 

In this present study, we investigated the possibility to use SMS from *P. eryngii* cultivation as a source of AFB1-degrading enzymes, with the aim to develop a new sustainable method for detoxification of AFB1-contaminated commodities. A crude extract was obtained from SMS (SMSE), which displayed Lac and MnP activity. In vitro trials were performed to optimize the conditions of the use of SMSE (temperature and pH) and maximize AFB1 degradation. The best conditions for the storage of SMS and SMSE were also investigated. To the best of the authors’ knowledge, there are no previous reports concerned with the use of crude extract from SMS for the removal of AFB1 from contaminated feed. 

## 2. Results

### 2.1. SMSE Enzymatic Activities 

In SMSE, the activity of two important ligninolytic enzymes, Lac and MnP, was determined by spectrophotometric assays. Among ten different SMS production batches, some variability in the enzymatic activity was observed. High Lac activity (4 U ± 1 g^−1^ of dry matter, DM) and low MnP activity (0.4 U ± 0.09 g^−1^ DM) were found in SMSE. The total protein content in SMSE was about 600 mg g^−1^ DM. 

### 2.2. Degradation of AFB1 by SMSE 

**Figure 1:**
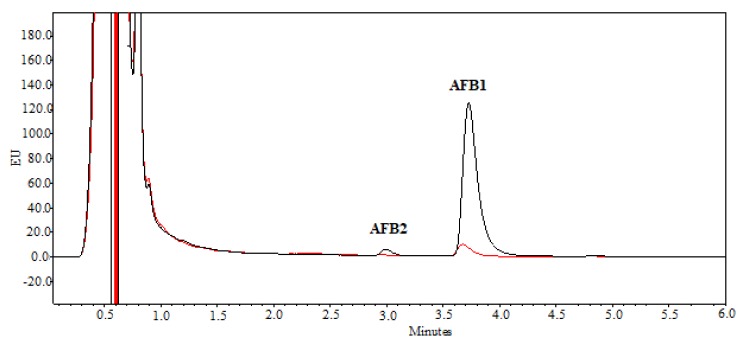
Comparison of UPLC/FLD (Ultra high performance liquid chromatography equipped with a fluorescence detector) chromatograms of aflatoxin B_1_ (AFB1) (885 ± 22 ng mL^−1^) and aflatoxin B_2_ (AFB2) in the positive control (black line), and in the sample after degradation by SMSE for 7 days at 25 °C (95 ± 2 ng mL^−1^ of AFB1, red line). Retention time of AFB1 = 3.7 min; retention time of AFB2 = 3.0 min.

AFB1 remained stable for 7 days when incubated in the extraction buffer and no significant variation in concentration (*p* < 0.05) was observed during this period of time (data not shown). The SMSE supplemented to AFB1 was able to determine the degradation of the mycotoxin in solution, as shown in Figure 1 by the reduction of the AFB1 UPLC/FLD (Ultra High Performance Liquid Chromatography equipped with a Fluorescence Detector) peak area of the SMSE treatment at the retention time of 3.7 min, corresponding to the AFB1 standard. The chromatographic peak at the retention time of 3 min in the positive control (Figure 1, black line) was aflatoxin B_2_ (AFB2), which was present as an impurity and was completely degraded by the treatment with SMSE (Figure 1, red line). No other peak is highlighted in the obtained chromatograms. No significant variation in the proportion of AFB1 degradation by SMSE was observed at increasing concentration of AFB1, in the range 154 ± 3–885 ± 22 ng mL^−1^ (Table 1); therefore, subsequent experiments were performed using the single dose of 885 ± 22 ng mL^−1^ of AFB1.

**Figure 2:**
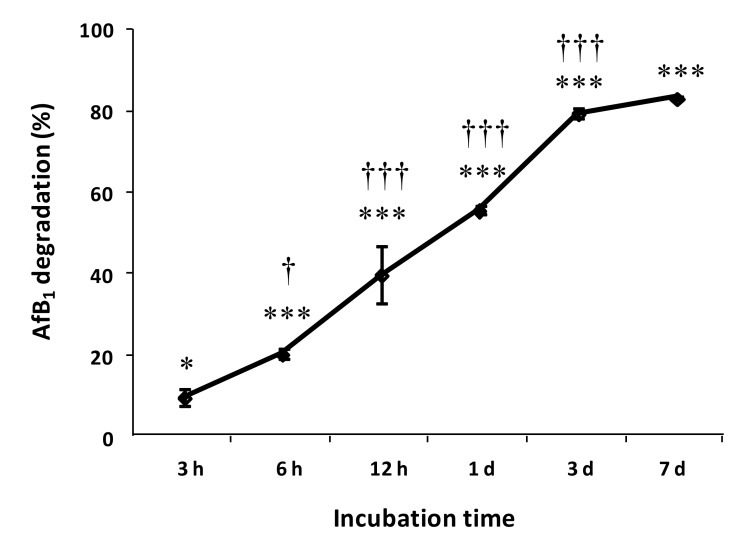
Time course of AFB1 degradation by SMSE. Data are expressed as mean ± SD (*n* = 3) of the percent AFB1 degradation with respect the control (AFB1 without SMSE). Statistically significant differences with the control by ANOVA are indicated by asterisks: * for *p* < 0.05 and *** for *p* < 0.001. A statistically significant difference with the previous value by Tukey–Kramer Multiple Comparison Test is indicated by daggers: † for *p* < 0.05 and ††† for *p* < 0.001.

The time course of AFB1 degradation by SMSE is shown in Figure 2. More than 50% AFB1 degradation occurred within 1 day of incubation, whereas after 3 days of incubation, the degradation percentage raised to 80% and did not change significantly from there on, till the 7th day of incubation. Based on these results, the maximum incubation time for the following experiments was 3 days.

**Figure 3:**
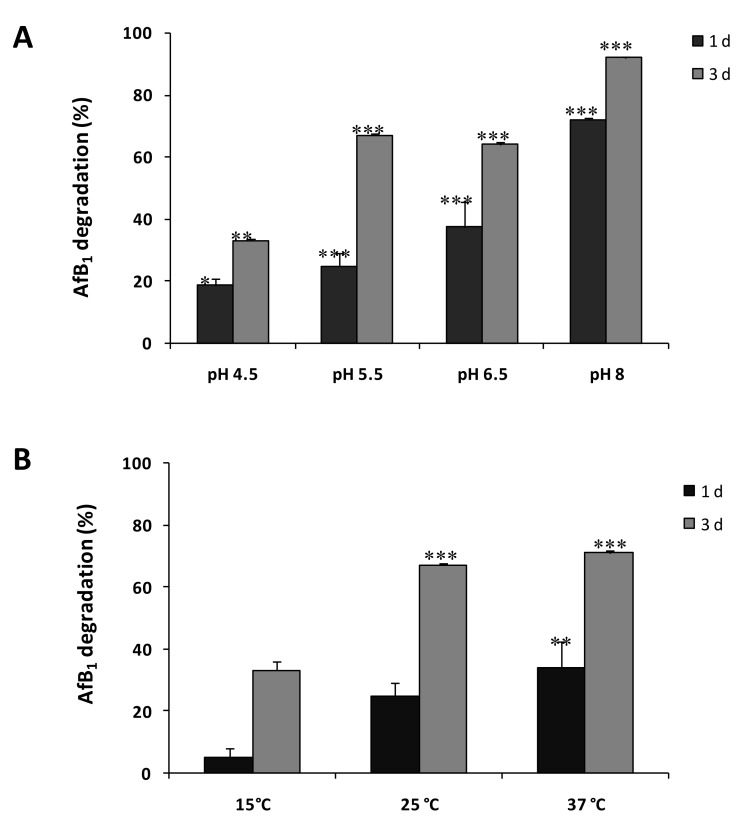
Effect of pH (**A**) and temperature (**B**) on AFB1 degradation by SMSE after 1 and 3 days of incubation. Data are expressed as mean ± SD (*n* = 3) of the percent AFB1 degradation with respect the control (AFB1 without SMSE). Statistically significant differences with the control by ANOVA are indicated by asterisks: * for *p* < 0.05, ** for *p* < 0.01 and *** for *p* < 0.001; Tukey–Kramer Multiple Comparisons Test.

The effect of temperature and pH on SMSE ability to degrade AFB1 is shown in Figure 3A,B, respectively. The results show that at temperatures between 25 and 37 °C, about half of AFB1 degradation occurred after 1 day of incubation, while the maximum degradation (80%) was reached after 3 days. When the temperature was lowered to 15 °C, the degradation was inhibited, reaching a maximum value of 25% after 3 days, and no degradation was observed after 1 day. The pH greatly influenced the degradative capability of SMSE. In particular, AFB1 degradation increased with the rise of pH from pH 4.5 to pH 8, exceeding at pH 8 the percentage of 70% and 90%, respectively after 1 and 3 days of incubation (Figure 3).

**Figure 4:**
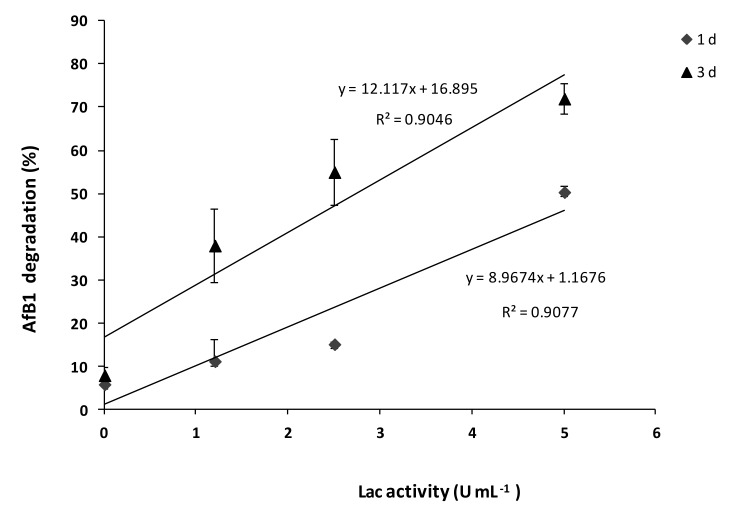
Scatterplot and fitted line showing the correlation between the laccase (Lac) activity of SMSE and AFB1 degradation, after 1 and 3 d of incubation at 25 °C. Data are express as mean ± SD (*n* = 3) of the percent AFB1 degradation with respect the control (AFB1 without SMSE). One Lac unit was defined as the quantity of enzyme able to oxidize 1 μmol of ABTS in a min, given a molar extinction coefficient ε_420_ = 3.6 × 10^4^ M^−1^ cm^−1^.

The degradative ability of SMSE was positively correlated with Lac activity (Figure 4); however, it was not possible to evaluate its correlation with MnP, due to the very low enzyme activity. The correlation analysis of data showed a strong relationship between Lac activity and AFB1 degradation, with R Squared values higher than 0.90 both at 1and 3 days of incubation (Figure 4). In particular, it was observed that in the presence of 5 U mL^−1^ of Lac, the percentages of AFB1 degradation were 50% and 70% after 1 and 3 days, respectively. Conversely, SMSE containing 2.5 and 1.25 U mL^−1^ of Lac degraded AFB1, respectively, by 50% and less than 40% after 3 days of incubation.

### 2.3. Storability of SMS 

**Figure 5:**
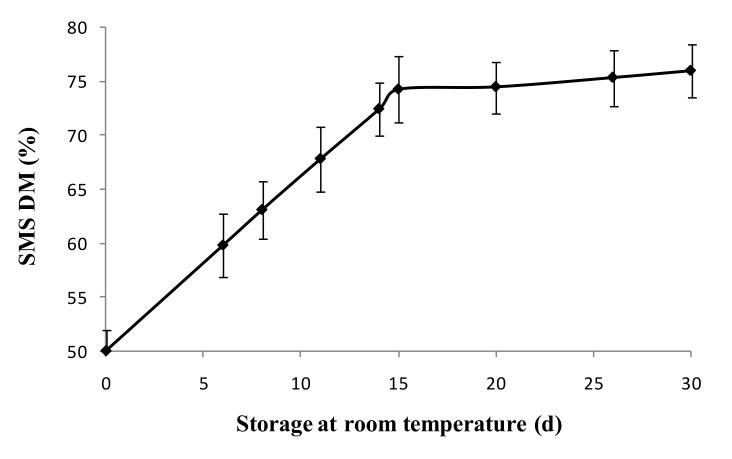
Percentage of dry matter (DM %, *w*/*w*) in SMS stored at room temperature. Data are expressed as means ± SD (*n* = 6).

Considerable variation in DM of different batches of newly produced SMS was found, with average values of about 50% and minus or plus variations of about 10%, depending on the place, period, and method of production. SMS stored at room temperature showed a progressive loss of water with a consequent increase of DM by about 25% in 15 days of storage. Subsequently, no significant variations of DM% were observed from there on, even after 30 days of storage (Figure 5). The loss of Lac activity of SMS stored at room temperature showed the same trend of the water loss, with a reduction of Lac activity by 30% (from 2.65 to 1.7 U g^−1^ DM) within 15 days and no further loss up to 30 days of storage. 

SMS stored in refrigeration at +4 °C did not show significant loss of Lac activity over 6 months of storage (data not shown).

### 2.4. Storability of SMSE

**Figure 6:**
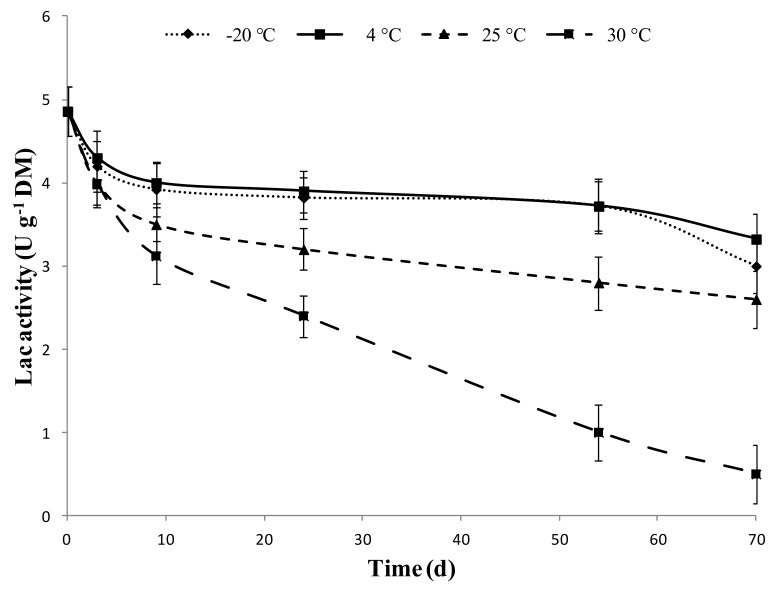
Effect of SMSE conservation at different temperatures (−20 °C, 4 °C, 25 °C, 30 °C) on Lac activity. Data are expressed as means ± SD (*n* = 3).

The effect of storage temperature on Lac activity of SMSE over a 70-day long period is shown in Figure 6. When stored at +30 °C, the Lac activity of SMSE decreased by 49% after 30 days and further decreased by 90% after 70 days of storage. At +25 °C, the loss of activity was less severe, but still high, with a decline of 34% and 47% of initial activity after 30 and 70 days, respectively. Furthermore, both at +25 °C and +30 °C storage temperatures, the development of molds in the extract was observed. On the contrary, storage under refrigerated (+4 °C) or freezing (−20 °C) conditions preserved 68% or 62%, respectively, of Lac activity over 70 days of storage. After lyophilization, Lac activity of SMSE decreased by 57%.

## 3. Discussion

As previously reported, *Pleurotus* spp. are efficient producers of ligninolytic enzymes, mainly laccase (Lac) and manganese peroxidase (MnP), whose application for aflatoxin degradation has been proposed [23,24,40]. The species *P. eryngii* (king oyster mushroom) has been shown to produce and release AFB1-degrading enzymes in the substrate used for mushroom cultivation [19,35]. The spent mushroom substrate (SMS) that remains as a waste after harvest, at the end of production cycle of the king oyster mushroom, is therefore a potential low-cost source of enzymes for biodegradation of aflatoxin and bioremediation of AFB1-contaminated commodities. With the aim to develop a new “green” method for the detoxification of aflatoxin-contaminated feed, we investigated the AFB1-degrading activity of an extract of SMS (SMSE) obtained from king oyster mushroom cultivation. 

In SMS of *P. eryngii* obtained from a mushroom farm, we found high levels of Lac activity and low levels of MnP activity, consistent with the findings of Li et al. [35]. The correlation that we found between Lac activity and AFB1 degradation by SMSE suggests that Lac is a major factor in AFB1 degradation, even if the presence of other catalysts or catalysis mediators is conceivable; in fact, Loi et al. [25] reported that a pure Lac from *P. pulmonarius* oxidized AFB1 with low efficiency, whereas it was able to degrade up to 73% of AFB1 when combined with mediator systems. Consistently, Li et al. [35] found that biodegradation of polycyclic aromatic hydrocarbons was higher with the use of crude extracts from *P. eryngii* than that with pure Lac.

The AFB1-degrading capability of *P. eryngii* SMSE, which in the optimal conditions of pH 8 and temperatures between 25 and 37 °C reached 70% and 90% after 1 and 3 day of incubation, appears noteworthy, when compared to the performances reported for other fungal or plant extracts or for purified laccases from other sources. Filtered cultures of *T. versicolor* containing 1.8 U mL^−1^ of Lac were able to degrade approx. 30% and 50% of AFB1 in solution at 30 °C, at the same incubation times of 24 and 72 h used in the present study [41]. An aqueous extract from the aromatic seed spice ajowan (*Trachyspermum ammi*) was reported to inactivate approximately 50% and 75% at 26 °C, and 75% and 90% at 37 °C of a mixture of aflatoxins B_1_, B_2_, G_1_, and G_2_ in 24 and 72 h, respectively, by an unidentified mechanism [42]. Alberts et al. [24] obtained 87% degradation of AFB1 in 72 h at 30 °C by incubating with 1 U mL^−1^ of a pure Lac from *T. versicolor.* A pure laccase enzyme (Lac2) from *P. pulmonarius* was tested by Loi et al. [40] for AFB1 degradation. However, the degradation achieved by incubating for 72 h at 30 °C with 2.5 U of Lac2 without redox mediators (namely 2,20-azino-bis-(3-ethylbenzothiazoline-6-sulfonic acid), acetosyringone, and syringaldehyde) was low, accounting for just 23% reduction of AFB1 content. With respect to the above-proposed applications, the technology that we experimented appears advantageous under various aspects. It does not require the dedicated cultivation of ligninolytic fungi in culture media to obtain active filtrates or extracts but, on the contrary, it relies on the recycling of disposable agricultural waste that, in an environment-friendly fashion, is thus transformed into a valuable by-product of mushroom cultivation. The production of SMSE is also a low-cost process and more economically sustainable than the use of purified enzymes, especially for developing countries and in marginal economies, where consumption of AFB1-contaminated foods and feeds has the highest social impact. Moreover, the possibility to store for long periods SMS (one month at room temperature) and SMSE (two months in refrigerated conditions) makes this waste and its raw extract particularly suitable for technological applications.

While the identification of the by-products and their toxicological characterization are gaps in the knowledge of chemical and microbial degradation of AFB1 that need to be bridged to implement these technologies into practical control measures, in the large majority of studies in this field, the degradation products still have to be identified. Branà et al. [19] analyzed the SMS of a laboratory-scale *P. eryngii* cultivation where 86% of the AFB1 had been degraded and did not find detectable levels of aflatoxicol, the main toxic metabolite of AFB1. An approach based on the cytotoxicity or mutagenicity testing of enzyme-treated materials has been used to claim the cleavage of AFB1 into non-toxic, or at least less toxic, fragments by laccases and other enzymes from various fungal or bacterial sources [17,24,41,43,44]. Alberts et al. [24] reported no significant differences in the mutagenic response of AFB1-containing samples treated with 1 U mL^−1^ of a pure Lac from *T. versicolor* with respect to negative control samples, in the *Salmonella typhimurium* mutagenicity assay. The treatment of 150 ng of AFB1 with a culture filtrate having laccase activity of 3.5 U mL^−1^ for 72 h at 25 °C also resulted in the complete loss of cytotoxicity on two human lymphoma cancer cell lines [41].

Several reports on the detoxification of feed- or foodstuff by treatments with water solutions of detoxifying agents can be found in the literature. Aqueous treatments with citric acid were used to reduce AFB1 in contaminated maize [45] as well as in extruded sorghum [46]. Scarpari et al. [41] sprayed AFB1-contaminated maize with culture filtrates of *T. versicolor* containing Lac and obtained 50% degradation with 3.5 U and 70% degradation with 7.0 U of the enzyme in 48 h. Recently, a similar approach has been used for the enzymatic detoxifying of maize from fumonisin mycotoxins [47] and the technology has been proposed to be utilized as a safer alternative to water washing that is practiced in subsistence farming communities in Africa for the treatment of whole maize intended for human consumption. A major advantage of the proposed technology was that the bulk solution of the residual enzyme and the less toxic degradation products could be easily separated from the treated maize kernels. A similar application is conceivable also for the degradation of AFB1 with SMSE, that qualifies as a simple, eco-friendly, low-cost, and efficient technology for remediation mainly of contaminated feedstuff, particularly in marginal areas. As previously noted by van der Westhuizen et al. [48]: “In many Sub-Saharan countries, where both maize contamination and maize consumption are high, regulatory mechanisms to control mycotoxin levels (…) are either lacking or are not enforced. Therefore, reducing exposure levels by intervention, specifically those based on simple low-cost measures acceptable to these communities, becomes critical to protect the population at greatest risk”. 

## 4. Conclusions

Based on these preliminary results, SMS from *P. eryngii* proved to be a suitable source of ligninolytic enzymes and in particular of Lac, a “green catalyst” able to degrade AFB1. The crude extract from SMS is easy to be obtained, not toxic, inexpensive, eco-friendly, and is able to remove up to 90% of AFB1. Further research is needed to confirm the absence of by-products with any residual toxicity. More studies are also needed to identify and fine-tune practical applications of SMSE to reduce AFB1 levels in animal feeds. In particular, “in vivo” tests on the major commodities affected by AFB1 are needed to set the most suitable soaking times and conditions and to experiment with different methods of SMSE application, such as spraying and washing techniques. 

## 5. Materials and Methods

### 5.1. Chemicals

AFB1 chemical standard (purity > 99%) and 2,2′-azino-bis 3-ethylbenzothiazoline-6-sulphonate (ABTS) were supplied by Sigma-Aldrich (Milan, Italy). All solvents (HPLC grade) were purchased from VWR International Srl (Milan, Italy). Water Millipore Milli-Q system (Millipore, Bedford, MA, USA) and RC 0.2 µm (regenerated cellulose membranes) filters were obtained from Grace (Deerfield, IL, USA). The SMS was supplied by the mushroom farm De Biase s.r.l. (Castellaneta, Italy).

### 5.2. Enzymes Extraction 

For extraction of the enzymes, a portion of SMS was taken in order to obtain a representative sample that included both the internal and external parts of SMS balls. Preliminarily, several extraction buffers were compared for efficient recovery of ligninolytic enzymes from SMS in an eco-friendly manner and at a low cost (data not shown). Finally, SMS crude extracts (SMSE) were obtained by blending 50 g of wet SMS with 150 mL of 0.1 M sodium phosphate (pH 7.3) at high speed for 3 min with a Sorvall Omnimixer. The extract was filtered with cheesecloth and centrifuged at 14,000 rpm for 15 min and then filtered through 0.22 μm pore size cellulose acetate filters (Sartorius AG, Muggiò, Italy). The enzyme activities (Lac and MnP) and total protein content of the resultant supernatants were estimated as described below.

### 5.3. SMSE Enzymatic Activities

Lac (EC 1.10.3.2) activity in SMSE was spectrophotometrically determined by oxidation of 2,2′-azino-bis 3-ethylbenzothiazoline-6-sulphonate (ABTS) at 37 °C. The reaction was performed in 100 mM of sodium malonate buffer (pH 4.5), 2 mM ABTS, and an appropriate amount of enzyme solution in a final volume of 1.5 mL. The increase in absorbance at 420 nm was evaluated with a spectrophotometer (Varian Cary 50, Agilent Technologies, Santa Clara, CA, United States. The enzymatic activity was determined by the molar extinction coefficient of ABTS (ε = 3.6 × 10^4^ M^−1^ cm^−1^). One unit (U) of Lac was defined as the amount of enzyme able to oxidize 1 μmole ABTS min^−1^ [49].

MnP (EC 1.11.1.13) activity was assayed by the oxidation of 1.0 mM MnSO_4_ in 50 mM of sodium malonate buffer, pH 4.5, in the presence of 0.05 mM H_2_O_2_. Manganese ions (Mn^3+^) form a complex with malonate, which absorbs at 270 nm (ε = 7.8 × 10^3^ M^−1^ cm^−1^). One unit of MnP is defined as the amount of enzyme producing 1 μmole of product per minute under the assay condition [49]. To increase Lac activity, SMSE was concentrated by ultrafiltration (AMICON YM1, cutoff size 10 kDa). Protein concentration was determined by the Bradford method, using bovine serum albumin as standard [50].

### 5.4. AFB1 Determination 

The standard solution of AFB1 was prepared by dissolving the commercial toxin (Sigma-Aldrich, Milan, Italy) in toluene/acetonitrile (9:1, *v*/*v*) into amber silanized vials to obtain a 1 mg mL^−1^ solution. The exact concentration of aflatoxin solution was spectrophotometrically determined according to AOAC Official Method 971.22 [51]. The residue was dissolved with water/methanol (60:40, *v*/*v*) to obtain calibrant standard solutions with 0.2, 0.4, 1.2, 2.0, 4.0, 5.0, and 10.0 ng mL^−1^ of AFB1. Standard solutions were stored at −20°C and warmed to room temperature before use. 

The UPLC apparatus was an Acquity UPLC system (Waters, Milford, MA, USA). Data acquisition and instrument control were performed by Empower software version 2 (Waters, Milford, MA, USA). The column used was a 100 mm × 2.1 mm i.d., 1.7 μm, Acquity UPLC^®^ BEH RP-18, with an Acquity UPLC column in-line filter (0.2 μm). AFB1 was detected by a fluorometric detector without postcolumn derivatization.

The fluorometric detector was set at wavelengths of 365 nm (excitation) and 435 nm (emission). The mobile phase was a mixture of water/acetonitrile/methanol (64:18:18, *v*/*v*/*v*) at a flow rate of 0.4 mL min^−1^. The temperature of the column was maintained at 40 °C. AFB1 was quantified by measuring the peak areas at the retention time of AFB1 standard and comparing these areas with the calibration curve of AFB1 in the range from 0.2 to 10.0 ng mL^−1^. With this mobile phase, the retention time of AFB1 was about 3.7 min. The limit of quantification (LOQ) of the method was 0.2 ng mL^−1^ for AFB1, based on a signal-to-noise ratio of 10:1.

### 5.5. Degradation of AFB1 by P. eryngii SMSE 

The AFB1-degradative ability of SMSE was tested for varying Lac activities, times, and conditions of incubation and the percent degradation of AFB1 was determined for the AFB1 theoretic concentrations of 125, 250, 500, and 1000 ng mL^−1^, which corresponded to UPLC/FLD-quantified concentrations of 154 ± 3, 322 ± 8, 462 ± 11, and 885 ± 22.

The experiments were performed in 1.5 mL Eppendorf-tubes in a final volume of 1 mL. The assay mixture contained 995 μL of SMSE and 5 μL of AFB1 solution. In control samples, the SMSE was replaced by an equal volume of extraction buffer. The degradation was conducted in the dark at 25 °C under continuous shaking at 120 rpm for 3, 6, 12, 24, 72, and 168 h. The effect of temperature (15, 25, 37 °C) and pH (4.5, 5.5, 6.5, 8) on SMSE degradative activity was also studied. The pH range was obtained by adding suitable amounts of concentrated HCl or NaOH solutions to reach the required pH values. To determine the AFB1 degradation obtained with varying Lac activities (5, 2.5, 1.2 U mL^−1^), the SMSE was diluted with sterile extraction buffer and incubated with AFB1 in the same conditions described above. All the experiments were performed in triplicate. 

After the enzymatic digestion, 500 μL of each sample were diluted with 500 µL of ultrapure water produced by a Milli-Q system, filtered through 0.2-µm-pore-size regenerated cellulose (RC) filters and 10 µL of the filtrate were injected directly into the UPLC apparatus through a full loop injection system. The percent degradation (D) was calculated by the formula D (%) = (C_f_/C_i_) × 100, where C_f_ was the concentration of AFB1 in the treatment with SMSE and C_i_ was the concentration of AFB1 in the control.

### 5.6. Storability of SMS

The persistence of Lac activity in stored SMS was assessed over a 30-day long period of time as a function of desiccation and temperature of storage (room temperature or +4 °C). Desiccation of SMS at room temperature was assessed by the percentage of dry matter over the total fresh weight (DM%). About 50 g of fresh SMS were precisely weighted to the nearest 0.01 g and then dried in a forced ventilation oven at 65 °C until a constant weight to determine the dry weight. The dry matter percentage was calculated as: DM% = dry weight/fresh weight × 100. SMS was stored on a shelf at room temperature or under refrigeration in a cool room at +4 °C for 30 days and then extracted and assayed for Lac activity as previously described. The experiments were carried out with six replicates per treatment.

### 5.7. Storability of SMSE

SMSE was obtained as previously described and stored at +30, +25, +4, and −20 °C for 70 days. SMSE was sampled at days 0, 3, 9, 24, 54, and 70 to determine the Lac activity as previously described. Moreover, the possibility to lyophilize the SMSE was also evaluated. The experiment was carried out with three replicates per treatment.

### 5.8. Statistical Analysis

Data were analyzed by one-way analysis of variance (ANOVA) and Tukey–Kramer multiple comparison test. The statistical analyses were performed using the GraphPad Instat 3.0 software (GraphPad Software, San Diego, CA, USA).

## Figures and Tables

**Table 1:** Percentage of AFB1 degradation (D) by SMSE at increasing doses of the mycotoxin (154 ± 3, 322 ± 8, 462 ± 11, 885 ± 22 ng mL^−1^) after 3 d incubation. Controls were incubated in the extraction buffer. Data are expressed as mean ± SD (*n* = 3).

Control AFB1 (ng mL^−1^)	SMSE	
	AFB1 (ng mL^−1^) ^(a)^	D (%) ^(b)^
154 ± 3	19 ± 4 *	88 a
322 ± 8	36 ± 2 *	89 a
462 ± 11	61 ± 2 *	87 a
885 ± 22	146 ± 2 *	84 a

^(a)^ Concentration of AFB1 found in SMSE at the end of the incubation time. Values in column followed by the asterisk are significantly different for *p* < 0.001 from the respective control (Tukey–Kramer Multiple Comparison Test). ^(b)^ Values in column followed by the same letter are not significantly different for *p* < 0.05 (Tukey–Kramer Multiple Comparison Test).
